# Oxidized Substrates of APEH as a Tool to Study the Endoprotease Activity of the Enzyme

**DOI:** 10.3390/ijms23010443

**Published:** 2021-12-31

**Authors:** Annamaria Sandomenico, Marta Gogliettino, Emanuela Iaccarino, Carmela Fusco, Andrea Caporale, Menotti Ruvo, Gianna Palmieri, Ennio Cocca

**Affiliations:** 1 Institute of Biostructure and Bioimaging, National Research Council (CNR-IBB), 80134 Napoli, Italy; annamaria.sandomenico@cnr.it (A.S.); emanuela.iaccarino@gmail.com (E.I.); andrea.caporale@cnr.it (A.C.); 2 Institute of Biosciences and BioResources, National Research Council (CNR-IBBR), 80131 Napoli, Italy; marta.gogliettino@ibbr.cnr.it (M.G.); carmela.fusco@ibbr.cnr.it (C.F.); ennio.cocca@ibbr.cnr.it (E.C.); 3 Department of Medicine and Health Sciences, University of Molise, 86100 Campobasso, Italy

**Keywords:** APEH, oxidized methionine, oxidized substrates, endoproteolytic activity, oxidative stress

## Abstract

APEH is a ubiquitous and cytosolic serine protease belonging to the prolyl oligopeptidase (POP) family, playing a critical role in the processes of degradation of proteins through both exo- and endopeptidase events. Endopeptidase activity has been associated with protein oxidation; however, the actual mechanisms have yet to be elucidated. We show that a synthetic fragment of GDF11 spanning the region 48–64 acquires sensitivity to the endopeptidase activity of APEH only when the methionines are transformed into the corresponding sulphoxide derivatives. The data suggest that the presence of sulphoxide-modified methionines is an important prerequisite for the substrates to be processed by APEH and that the residue is crucial for switching the enzyme activity from exo- to endoprotease. The cleavage occurs on residues placed on the C-terminal side of Met(O), with an efficiency depending on the methionine adjacent residues, which thereby may play a crucial role in driving and modulating APEH endoprotease activity.

## 1. Introduction

The paradox of aerobic life is that higher eukaryotic organisms cannot exist without oxygen, but it is potentially dangerous to their existence. This ‘dark side’ of oxygen is related to the reductive environment of the cellular milieu, which provides many opportunities for it to undergo spontaneous reduction. Specifically, the sequential reduction of oxygen leads to the production of a number of free radicals, collectively called reactive oxygen species (ROS) that are more reactive than molecular oxygen and responsible for its toxicity. ROS are able to damage cellular macromolecules, strongly impairing their functional and structural efficiency. Therefore, living organisms have acquired several defense mechanisms to self-protect from oxidative damage, which if not properly regulated, may lead to aging and several related diseases and pathologies [1]. It is widely accepted that high ROS levels may have deleterious effects on macromolecules such as proteins and different proteolytic systems that operate in a number of different biochemical mechanisms aimed at tissue detoxification. In this context, recent studies assign to APEH (Acylaminoacyl Peptidase or acylamino-acid-releasing enzyme) a crucial role in these mechanisms, given its ability to process and degrade many protein substrates through both exo- and endopeptidase activity [2,3,4,5,6,7,8].

APEH is a ubiquitous and cytosolic serine protease belonging to the prolyl oligopeptidase (POP) family (clan SC, family S9) [9,10]. As such, it contains a peptidase domain with a α/β-hydrolase fold and an unusual β-propeller domain, which covers the catalytic triad [9,11]. From a physiological point of view, APEH plays a key role in protein metabolism by participating in amino acid recycling, as it catalyzes the hydrolysis of N-acetylated peptides/proteins yielding an N-acylated-amino acid and a peptide/protein with a free N-terminus [3,12]. Initially assumed to exclusively contribute to the protein turnover acting as exopeptidase, APEH was then demonstrated to have distinct functional properties to help organisms survive under adverse conditions, thus playing an important role in different biological processes. Specifically, APEH exhibited additional endopeptidase activity on oxidatively damaged or heavily glycated proteins [4,6,7,8,13,14], and thus it is referred as OPeH (oxidized protein hydrolase), thus coordinately contributing, with the proteasome, to the elimination of cytotoxic proteins and to fight proteotoxic stress [14,15]. Recently, it has been reported that APEH may translocate in the nucleus, where it is involved in single-stranded DNA repair and cell survival after exposure to H_2_O_2_, thus cooperating in the DNA damage response [16]. The emerging multifaceted role of APEH in the degradation of damaged proteins and in cell protection under oxidative stress conditions make it a potential therapeutic target for a wide array of human pathologies, ranging from aging to diseases such as Alzheimer’s, cancers, and diabetes, which are all caused by the accumulation of harmful proteins [13,17,18,19,20,21]. However, despite its biological importance, extensive studies on the specific biochemical role of APEH in antioxidant defense systems have not been addressed, although it would be crucial to better understand the molecular mechanisms involved and develop new therapeutic strategies.

Generally, one main difficulty in studying enzyme functions derives from their ability to work in dynamic living systems, and from their activity, which is modulated among others by post-translational modifications, protein-protein interactions, and endogenous inhibitors. Hence, exploring how APEH participates in the regulation of redox homeostasis is extremely challenging, and identifying novel tools such as small substrate molecules to obtain further insights into the ‘oxidation-induced’ endopeptidase activity of APEH in the biological context, is prominent. Thus far, despite the wide variety of existing substrates on the market to assay the exopeptidase activity of this enzyme, synthetic chemical probes for endoproteolytic activity are not yet available. 

In this study, a set of model peptides deriving from the GDF11 (growth differentiation factor 11) was designed ad hoc with the aim to be used as model substrates to investigate the endoproteolytic activity of APEH. These peptides were chosen due to the presence of residues (methionine, histidine and tyrosine) susceptible to oxidation by endogenous or exogenous chemicals [22]. 

For this reason, these compounds can represent useful models to gain mechanistic insights into substrate–enzyme recognition and the correlation between oxidation and acquisition of endoproteolytic activity toward oxidized substrates.

## 2. Results and Discussion

### 2.1. Preparation and Oxidation of Peptides

In recent years, peptide-based technologies have considerably expanded the ability to outline the substrate specificity of proteases, whose characterization represents a critical aspect to improve our knowledge on the enormous repertoire of cellular functions regulated by these enzymes both in physiological and pathological conditions. 

In this context, the peptide GDF11[48-64], here named GDF11[48-64]wt, reproducing the 48–64 region of GDF11 protein (growth differentiation factor 11), was selected as a model probe to perform this study, given the presence of two highly oxidizable methionines residues and other oxidation-prone amino acids such as histidine and tyrosine. 

GDF11[48-64]wt was converted to the variant with oxidized methionines by treatment with 3% H_2_O_2_, monitoring the reaction progression by reverse-phase HPLC chromatography (RP-HPLC) and mass spectrometry, using the un-oxidized precursor as control. The occurrence of the peroxide oxidation rendered the peptide more hydrophilic than its unoxidized counterpart, as clearly indicated in Supplementary Figure S1A,B by the shift in the RP-HPLC mobility of GDF11[48-64]ox to shorter retention time (9.97 min) with respect to that of GDF11[48-64]wt (11.41 min). Following oxidation, the observed molecular mass (2223.03 amu) of GDF11[48-64]ox (Supplementary Figure S1D) increased by 32 amu with respect to that of the native peptide (2191.04 Da) (Supplementary Figure S1C), strongly indicating that two oxygen atoms were added after H_2_O_2_ treatment.

**Figure 1:**
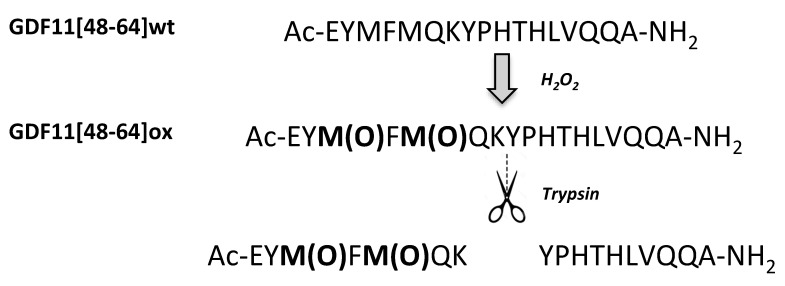
Scheme of the hydrolysis of GDF11[48-64]ox by trypsin and identification of the oxidation sites on methionines.

**Figure 2:**
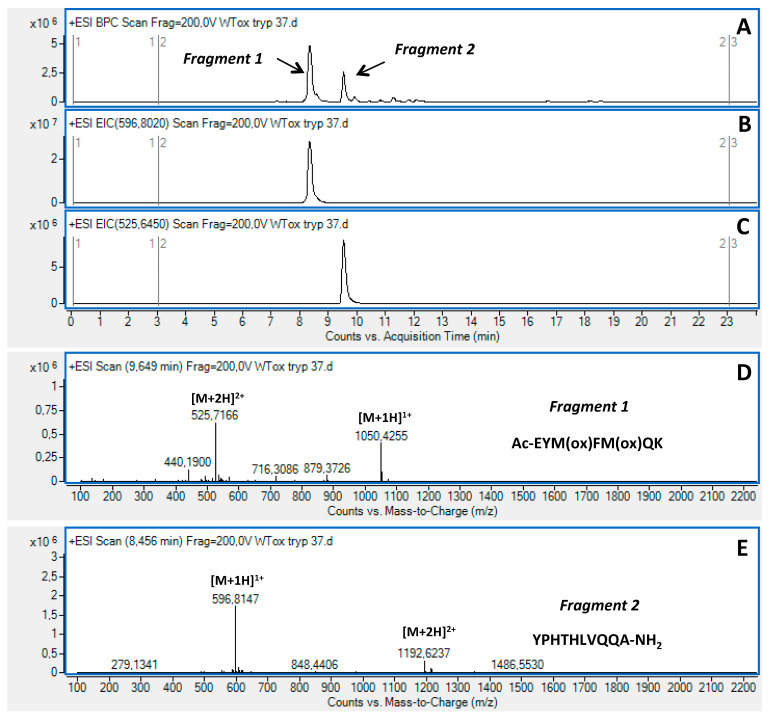
LC-MS analysis of GDF11[48-64]ox following tryptic digestion. Base peaks profiles of the peptide after oxidation and treatment with trypsin (**A**). EIC analysis of Fragment 1 (Ac-EYMFMQK), (**B**). EIC analysis of Fragment 2 (YPHTHLVQQA-NH2), (**C**). Mass spectrum of Fragment 1, (**D**). Mass spectrum of Fragment 2, (**E**).

However, given the presence in the GDF11[48-64]wt sequence of further residues such as histidine and tyrosine able to undergo oxidation, a tryptic digestion followed by LC-MS analysis was performed to assess the oxidation of methionines (Figure 1 and Figure 2A–E). The LC-MS analysis of the two fragments generated by the tryptic cleavage on K54, Ac-EYMFMQK (Fragment 1) and YPHTHLVQQA-NH2 (Fragment 2) strongly suggested that oxidation occurred at level of the two methionines, since Fragment 2 was found unaltered, while Fragment 1 showed a molecular mass increased by 32 amu. In addition, the oxidation of Y49 on Fragment 1 was excluded on the basis of the UV spectrum, which was unaltered before and after the oxidation treatment (data not shown, [23,24]. Following further analyses by extracted ion chromatograms (EICs), the occurrence of molecular species bearing further oxidations were also excluded (i.e., three oxidations, Δmass +48 amu; four oxidations Δmass +60 amu) on both the full peptide and the Fragment 1. The absence of a sulphone group on single methionines (Δmass +32 amu) was excluded by a comparative RP-HPLC analysis of a reference GDF11[48-64]ox prepared using Fmoc-Met(O)-OH and generated by peroxide oxidation (not shown). This analysis further confirmed the absence of the oxidation on Y49. We thus concluded that the only species generated by peroxide treatment were the molecules bearing sulphoxide groups on M50 and M52. All the other peptides reported in Table 1 except the GDF11[48-64]M50M52AA control peptide, were quantitatively converted to the expected Met-oxidized species and similarly characterized (data not shown). Peptides GDF11[48-64]wt, GDF11[48-64]M50M52AA, and GDF11[48-64]ox were also submitted to a structural analysis by circular dichroism (CD) spectroscopy to assess their structure in solution and investigate any correlation between conformation and susceptibility to APEH cleavage. As shown in Supplementary Figure S2, all peptides exhibited random conformations, thus suggesting that proneness to cleavage was independent from the structure. 

### 2.2. APEH Purification from Blood Red Cells (RBC)

Human APEH was obtained as a partially purified product from the red blood cells by a combination of anion exchange and gel filtration chromatography as described. The purification procedure was monitored by SDS-PAGE analysis of active pooled fractions (Supplementary Figure S3). Specifically, each peak fraction eluted from the gel filtration column was assayed with the specific APEH substrate Ac-Met-AMC and analyzed by Western blotting to assess the identity of the enzyme, using the commercial APEH from porcine liver (APEH_pl_) as control (Supplementary Figure S4A,B). The molecular mass determined by SDS-PAGE of the partially purified protein was 75 kDa, in agreement with that predicted on the basis of the amino acid sequence. Moreover, gel filtration chromatography on Superdex 200 column allowed estimating a molecular mass for the native APEH of ~280 kDa as calculated by the calibration curve (Supplementary Figure S4A), confirming that the enzyme is a homo-tetramer composed of four 75 kDa subunits, as already reported [9]. To further validate the enzyme preparation, an inhibition assay was performed using the potent and selective inhibitor AA74-1 showing that APEH activity was totally inhibited in the presence of the inhibitor, thus confirming its identity (not shown). 

### 2.3. Identification of the APEH Cleavage Sites on Oxidized GDF11[48-64] by LC-MS Analyses

In order to identify the potential cleavage sites of APEH on the oxidized GDF11[48-64], the peptide was incubated alone or in combination with the enzyme and the corresponding reaction mixtures were analyzed by LC-MS. The same experiments were performed using the non-oxidized analogues as control. 

**Figure 3:**
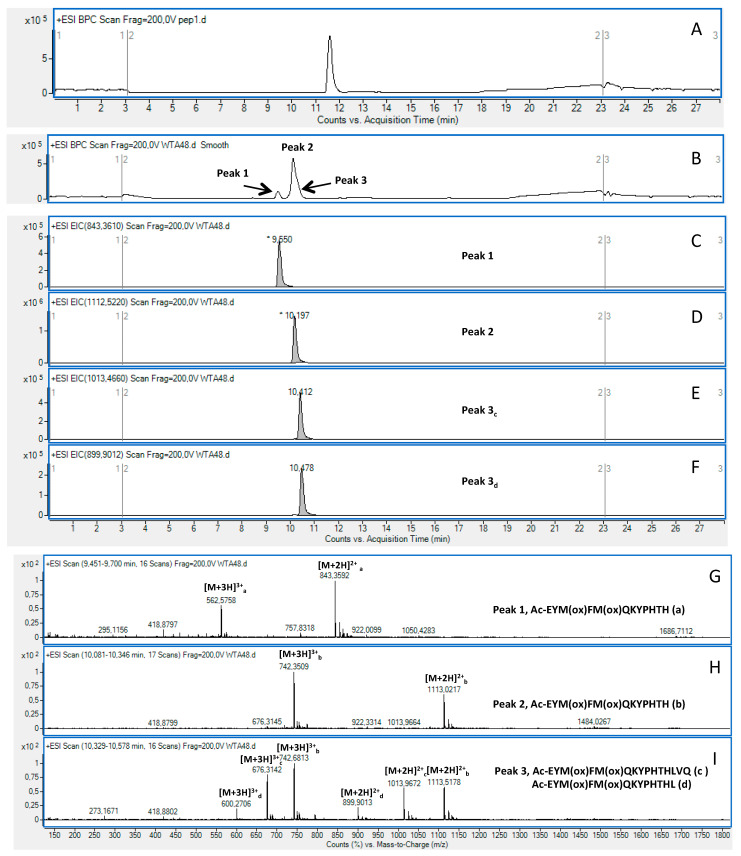
LC-MS analysis of the GDF11[48-64]ox before and after treatment with APEH for 48 h. Base Peak profiles of GDF11[48-64]ox (**A**) before and (**B**) after treatment with APEH where the 3 peaks generated are indicated by arrows. (**C**–**F**) EIC profiles of Peaks 1–3. (**F**–**I**) Mass spectra of Peak 1, Peak 2 and Peak 3. Under Peak 3, two main species were identified as the fragments Ac-EYM(ox)FM(ox)QKYPHTHLVQ (species c) and Ac-EYM(ox)FM(ox)QKYPHTHL (species d). The species identified are reported on the mass spectra.

In contrast to what observed with the non-oxidized peptide (Figure 3A), the analyses performed on the oxidized GDF11[48-64]wt after treatment with APEH evidenced the presence of two extra peaks in the RP-HPLC profile, one at 9.55 min (Peak 1) and the second one (Peak 3) at 10.41 min, which appeared as a shoulder of the main peak attributed to the intact molecule (Peak 2, retention time 10.19 min, Figure 3B). A detailed analysis of the EIC peak areas (Figure 3C–F) revealed that some 50% of the starting peptide was degraded by the enzyme, providing under Peak 3 the fragments: Ac-EYM(ox)FM(ox)QKYPHTHLVQ (denoted as Peak 3_c_) and Ac-EYM(ox)FM(ox)QKYPHTHL (denoted as Peak 3_d_) lacking, respectively, the QA and VQQA fragments at the C-terminus. Peak 3_c_ had a MW of 2024.930 amu and accounted for about 16% of the initial amount of molecule; Peak 3_d_ had a MW of 1797.802 amu accounting for about 8% of the initial molecule. Under Peak 1, the peptide Ac-EYM(ox)FM(ox)QKYPHTH, lacking the LVQQA portion at the C-terminus was identified (MW: 1686.715 Da) accounting for about 26% of the intact molecule) (Table 2a). No cleavage was observed when the GDF11[48-64]wt was in its non-oxidized form. These results suggested that the model peptide GDF11[48-64]wt, when converted into the doubly oxidized variant GDF11[48-64]ox, becomes prone to the endo-proteolytic degradation of APEH, which preferentially cleaves at H59 (around 50% of the total processed peptide) but also cleaves at Q62 (around 32% of the total processed peptide) and at L60 with only about 17% of the total processed peptide. 

Subsequent efforts were focused on exploring the effects of replacing some specific amino acids in GDF11[48-64]wt sequence on the susceptibility of GDF11[48-64]wt to the APEH activity. The set of GDF11[48-64]wt analogues, designed as reported in Methods section (Table 1), was thus prepared, oxidized and analyzed by LC-MS following exposure to APEH. 

As reported in Table 2, APEH exhibited endoprotease activity toward all variants following the peroxide-mediated conversion of methionines to methionine sulfoxide, and all of them were cleaved on the carboxy side of H59 and Q62, similar to the parent molecule. In addition, the molecules were preferentially split on the carboxy side of H59 as compared to Q62, with a percentage of H59 cleavage ranging from about 11% (Y49A and M50F51AA mutants) to 28% (GDF11 wt). Instead, the proteolysis on Q62 varied from 0.3% (on F51M52AA double mutant) to 18% (GDF11 wt). APEH showed a similar specificity for Q62 (10.2% cleavage) and H59 (11.5% cleavage) sites in the mutant Y49A (Table 2). Of note, the K54E mutant did not undergo cleavage on Q62, indicating that replacement of lysine with a residue having an oppositely charged side chain as glutamic acid (E) completely shifted the cleavage specificity of the enzyme toward H59 (18.7%, Table 2).

**Figure 4:**
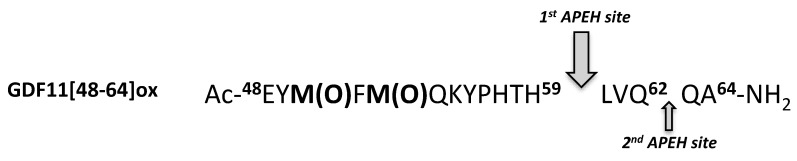
Scheme of main cleavage site of APEH on GDF11[48-64]ox.

Remarkable differences were also observed on the total amounts of processed peptides, which varied from about 50% (wt peptide) to about 13% (M50F51AA) (Table 2). Taken together, these data suggest that APEH exerts endoproteolytic activity on peptide substrates bearing sulphoxide-modified methionines, whose presence is an important pre-requisite for substrate recognition and for switching the enzyme activity from exo- to endoprotease. Data suggest also that proteolysis occurs on residues placed on the C-terminal side of Met(O), with an efficiency depending on the methionine adjacent residues, which thereby play a crucial role in driving and modulating the APEH endoprotease activity. Moreover, on the GDF11[48-64]ox substrate, the cleavage preferentially occurs at a distance of 7 or 9 residues from the first or second Met(O), respectively, and amino acids close to them, including Y49, F51 and K54, are able to critically influence the cleavage rate on the first and second site (Figure 4).

### 2.4. Comparative Analysis of the Cleavage Sites of APEH toward Different Oxidized Substrates

It is worth comparing the APEH cleavage site motif identified in our model peptide with those found in substrates already reported in the literature. Until now, three APEH endoprotease substrates have been characterized as: the Insulin A Chain (processed by a truncated form of APEH) [25,26,27,28] and the β-amyloid peptide. The four sequences thus far investigated and the relative APEH cleavage sites are reported in Table 3.

As shown, the lack of a well-defined consensus motif among the four sequences is evident. This finding is not surprising considering that the endoproteolytic activity of APEH is targeted toward oxidatively damaged proteins, and therefore it cannot depend on the presence of rigid consensus motifs. Conversely, further structural factors must be taken into account, making these substrates recognizable by the enzyme and subjected to the proteolytic cleavage. In Table 3, the oxidized and experimentally verified amino acids are highlighted, as well as all those considered most susceptible [26] to oxidative damage, i.e., Cys (C), Met (M), and the four aromatic amino acid residues Phe (F), His (H), Trp (W), Tyr (Y). However, despite the lack of a consensus sequence, it is interesting to underline that the endoproteolytic cleavage sites of APEH are often adjacent or near to oxidized or more oxidizable residues. In this context, the most representative is the β-amyloid peptide, in which the three cleavage sites are all proximal to oxidized residues [27,28]. As the oxidation of amino acids gives rise to the formation of negative charges, it is reasonable to assume that this chemical modification could be crucial for the substrate-APEH recognition when it works as an endoprotease, through a striking clamshell-like inter-lobe dynamics for the substrate selection [29].

It has been reported that the native conformation of the enzyme can be described as a dynamic equilibrium between open and closed states, and the opening–closing mechanism has been suggested to drive the substrate screening. Generally, the open state is associated with a functionally disabled isoform although bulkier substrate candidates may bind to the catalytically impaired but available active site, inducing the closure of the two lobes. This model could provide a possible mechanism of recognition of oxidized substrates by APEH based on their size and structural complexity. The structure of the protein has been recently predicted with AlphaFold, the most reliable free server for generating accurate protein structures, and it is reported in the main protein databases (see for example https://alphafold.ebi.ac.uk/entry/P13798, accessed last time on 23 December 2021, [30]). The structure is similar to that obtained experimentally for Aeropyrum pernix [31], showing the bi-lobate structure and the tunnel in the β-propeller domain that facilitates the access of substrates to catalytic site (see Supplementary Figure S5A–C).

## 3. Materials and Methods

All protected amino acids, including 7-N-Fmoc-aminocoumarin-4-acetic acid (Fmoc-ACA-OH), Fmoc-Met(O)-OH, the coupling agents (1-[Bis(dimethylamino)methylene]-1H-1,2,3-triazol [4,5-b]pyridinium 3-oxid-hexafluorophosphate (Hexafluorophosphate Azabenzotriazole Tetramethyl Uronium, HATU), diisopropylcarbodiimide (DIC), ethyl cyanohydroxyiminoacetate (OXYMA)) and Fmoc-Rink Amide AM Resin used for peptide synthesis were purchased from IRIS Biotech GmbH (Marktrewitz, DE). Acetonitrile (CH_3_CN), dimethylformamide (DMF), trifluoroacetic acid (TFA), and methanol (CH_3_OH) were purchased from ROMIL (Dublin, Ireland). Other products, such as Sym-collidine, N, N-Diisopropylethylamine (DIEA), piperidine, acetic anhydride, hydrogen peroxide 30% *v*/*v* (H_2_O_2_) and ethyl ether, were from Sigma-Aldrich (Milan, Italy). RP-HPLC analytical analyses were performed on a WATERS Alliance e2695 (WATERS, Milano, Italy) equipped with a WATERS 2998 PDA detector using ONYX monolithic C18 columns (50 × 2.0 mm ID) Phenomenex (Casalecchio sul Reno, Italy) running at a flow rate of 0.6 mL/ min. Peptide purifications were performed on a preparative WATERS 2545 Quaternary Gradient Module HPLC supplied with a WATERS 2489 UV–visible Detector using an XBRIDGE Prep BEH130 OBDTM C18 (5 μm, 50 × 19 mm ID) Waters column operated at a flow-rate of 10 mL/min. For preparative RP_HPLC separations, solvents were: Buffer A: 0.1% TFA in H_2_O and Buffer B: 0.1% TFA in CH_3_CN. LC-ESI-TOF-MS (LC-MS) analyses were performed with an Agilent 1290 Infinity LC System coupled to an Agilent 6230 time-of-flight (TOF) MS System (Agilent Technologies, Cernusco Sul Naviglio, Italy). The liquid chromatograph Agilent 1290 LC module was also coupled with a photodiode array detector (PDA) using a C18 Aeris column (3 µm, 4.5 × 50 mm) from Phenomenex operating at 0.20 mL/min. Solvents were: Buffer A: 0.05% TFA in H_2_O; Buffer B: 0.05% TFA in CH_3_CN.

### 3.1. Peptide Design

All peptide substrates used in this study were designed as amidated and acetylated products. The set of molecules is reported in Table 1 and includes GDF11[48-64] and some related mutants designed as described below. GDF11[48-64] was chosen as a starting model molecule since it contains several residues such as methionines, histidines and tyrosines, which are highly susceptible to chemical or enzymatic oxidation. In particular, the peptide bears on positions 50 and 52 two methionines, whose side chains can undergo oxidation by oxygen radicals to give the corresponding sulphoxides or sulphones. On positions 57 and 59, two histidines are present, which at a lesser extent, compared to methionines, are prone to oxidation by radicals, metal ions and other reagents [22,32,33] to mostly give 2-oxo-histidine. Tyrosines, occurring on positions 49 and 55 of the peptide, bear a phenol side chain also sensitive to oxidation by the same reagents but at a slower rate [22]. The set of GDF11 mutated peptides were thus designed considering that, being methionines most susceptible to oxidation [22], sensitivity to APEH endoprotease activity could be first acquired following modification of these residues. Ala-mutants were thus designed in order to replace one or both the methionine’s side chins with unreactive methyl groups and to assess whether the selective incorporation of oxygen atoms in methionines would impart to the peptide the ability to be cleaved somewhere downstream of these residues. Other single and double mutants were also designed and prepared to investigate the possible role played by residues adjacent to methionines, such as lysine 54 (K54), tyrosine 49 (Y49) and phenylalanine 51 (F51), on peptide sensitivity to APEH activity. The peptide GDF11[48-65]M50M52AA, where the two methionines were replaced with alanine residues, was used as a control peptide.

### 3.2. Synthesis and Purification of Peptides

All peptides used in this study were synthesized by the solid phase method as amidated and acetylated products and purified by RP-HPLC as reported elsewhere [34].

Peptides were manually assembled on 70 mg of Rink Amide AM resin (loading 0.71 mmol/g, synthesis scale 50 µmoles), performing single couplings (1h) using OXIMA/DIC or HATU/Sym-Collidine as activating agents. Fmoc deprotection from α-amino groups was achieved by a 2-step treatment with 40% piperidine in DMF for 5 min followed by a second treatment with 20% piperidine in DMF for 15 min. The GDF11[48-64] bearing the two Met(O) residues was similarly prepared and purified in order to obtain a reference for the GDF11[48-64]ox variant generated by peroxide treatment. Peptide resins were treated with a mixture of trifluoroacetic acid (TFA)/tri-isopropylsilane (TIS)/water (90:5:5) for 4 h to remove the side chains and the resin. After filtration, crude peptides were recovered by precipitation with cold ethyl ether and lyophilized.

Following RP-HPLC purification, peptides were obtained in high yields and their identity and purity (>95%) was confirmed by LC-ESI-TOF analyses (Table 1). The final products were solubilized in 100 % DMSO at 10 mM and stored at −20 °C.

### 3.3. Oxidation of Peptides by H_2_O_2_ and Circular Dicroism (CD) Analyses

In order to obtain the variants with oxidized methionines, 2 mg aliquots of purified peptides (about 1 µmol) in 1.0 mL of water were spiked with 100 µL of 30% *v*/*v* H_2_O_2_ in water for 30 min. The final concentration of H_2_O_2_ was about 3%. Following RP-HPLC analyses to assess the complete conversion, peptide solutions were firstly acidified with TFA up to 0.1% and then purified to homogeneity by preparative RP-HPLC. Purified aliquots were lyophilized, confirming identity and purity by LC-ESI-TOF analyses. CD studies were performed on the peptides GDF11[48-64]wt, GDF11[48-64]M50M52AA, and oxidized GDF11[48-64]wt. Peptides were dissolved in water at 50 µM and spectra were recorded as reported elsewhere [35].

### 3.4. Tryptic Digestion

In order to verify which residues underwent oxidation following H_2_O_2_ treatment, GDF11[48-64] and the related mutants (excluding GDF11[48-64]K54E bearing a glutamic acid in place of a lysine) were treated for 2 h at 37 °C with TPCK-added trypsin (Sigma-Aldrich, Milano, Italy) at 1:100 enzyme:substrate (*w*/*w*) ratio. The mixtures were next analyzed by LC-ESI-TOF mass spectrometry.

### 3.5. Partial Purification of APEH from Human Red Blood Cells

Blood samples were taken from donors using syringes with heparin. In accordance with ethical principles stated in the Declaration of Helsinki, as well as with approved national and international guidelines for human research, a written informed consent was obtained from participants. The erythrocytes were separated from the plasma by centrifugation at 1800× *g* for 5 min at 4 °C and washed twice with an isotonic solution (10 mM Tris-HCl pH 7.6, 1.7% NaCl; 4 °C). Lysis of erythrocytes was carried out by incubation in hypotonic solution (25 mM Tris/HCl, pH 7.5) for 30 min on ice. The hemolysate was obtained by centrifugation of the lysate at 9200× *g* for 40 min at 4 °C. Then, the ‘soluble fraction’ was loaded onto a DEAE Sepharose Fast Flow column, connected to an AKTA FPLC system (Amersham Biosciences, Amersham, UK), previously equilibrated in 25 mM Tris-HCl (pH 7.5) (buffer A). The bound proteins were eluted using a linear ionic strength gradient (0–100%) of 1 M NaCl in buffer A at a flow rate of 1 mL min^−1^. The active fractions were collected, pooled, dialyzed against 25 mM Tris-HCl pH 7.5 and loaded on a Superdex 200 PC 3.2/30 column (Pharmacia Biotech, Stockholm, Sweden) connected to a SMART System (Pharmacia Biotech, Stockholm, Sweden). The bound proteins were eluted with 25 mM Tris-HCl buffer, pH 7.5, containing 50 mM NaCl, at a flow rate of 0.1 mL min^−1^. The active fractions were collected, pooled, dialyzed against 25 mM Tris-HCl pH 7.5 and stored in the same buffer supplemented with 5% glycerol. Enzyme purification was monitored using Ac-Met-7-amino-4-methylcoumarin (AMC) as substrate [6]. Active fractions were analyzed by SDS-PAGE (12%) in order to evaluate the enrichment of the protein during the various purification steps. The protein was also characterized by Western Blot analysis. Aliquots of protein samples were subjected to SDS-PAGE (8%), and then electroblotted onto PVDF membranes (ImmobilonTM, Millipore, Milan, Italy). Membranes were next incubated with the specific primary antibody (sc-102311, 1:5000, Santa Cruz Biotechnology, USA) and then with the horseradish peroxidase-conjugated secondary antibody for 1 h at 37 °C (1:5000, Santa Cruz Biotechnology, Dallas, TX, USA). The immune complexes formed were visualized by enhanced chemiluminescence and autoradiography, according to the manufacturer’s protocol (Amersham Biosciences, Amersham, UK). Molecular mass of the native enzyme was established by gel filtration chromatography on a Superdex 200 PC 3.2/30 column (Pharmacia Biotech, Stockholm, Sweden), connected to a SMART system (Pharmacia Biotech, Stockholm, Sweden), and calibrated with BioRad gel filtration standards (BioRad, Milano, Italy; code 151-1901). The enzyme inhibition assay was performed on APEH preparation using the selective and potent inhibitor AA74-1 (SML0358, Sigma-Aldrich, Milano, Italy). Mixtures containing appropriate amount of inhibitor (62 µM) and APEH sample (0.7 μg) were pre-incubated for 30 min at 37 °C; then the substrate Ac-Met-7-amino-4-methylcoumarin (AMC) was added to the assay mixture and the enzymatic activity was followed as previously described [6]. Control samples were prepared by pre-incubating the same amounts of APEH without the inhibitor and then assayed following the same procedure.

### 3.6. Treatment of Peptides with APEH and Assessment of Proteolysis by Mass Spectrometry

Treatment with APEH was performed on the peptides listed in Table 1 after oxidation. In total, 10 nmol/200 μL of each molecule was incubated with APEH partially purified from human blood cells (0.7 μg, 24.000 total units) at 37 °C for 48 h in Tris-HCl 25 mM pH 7.5. Proteolytic mixtures were analyzed by LC-MS using a C18 X-Bridge Waters column (3 μm, 4.6 × 50 mm) operating at 0.20 mL/min and applying a linear gradient of 0.05% TFA in acetonitrile (Solvent B; Solvent A was H_2_O, 0.05% TFA) in 20 min.

## 4. Conclusions

In the last years, APEH has received increasing attention due to its involvement in important cellular mechanisms of protein disposal [12,15,16,19,36]. However, its endoproteolytic activity, contributing to the elimination of proteins impaired by oxidative processes or other chemical modifications occurring in pathological events, has been poorly characterized. Therefore, the growing interest in better understanding the biological functions of APEH requires urgent development of not laborious and suitable in vitro assays that allows to specifically detect and quantify the activity of this protease. However, this is mostly hampered by the lack of information regarding the substrate’s structural requirements that drive the enzyme recognition and the sites undergoing to the endoproteolytic cleavages.

In our study, we found that APEH acquires the ability to bind and process peptide substrates only when they include oxidized methionines. The enzyme–substrate recognition event appears more efficient not only in the presence of multiple Met(O) sites but also in dependence of their optimal surrounding environment. Looking at the degradation rates obtained with the different substrates, we observed that the presence of Met(O) residues was a necessary but not sufficient pre-requisite for triggering efficient endoproteolytic cleavage. Our findings suggest that the negatively charged side chains of the oxidized methionines are likely to be the docking point for the enzyme, while the flanking residues can drive the proteolytic reaction.

To the best of our knowledge, this is one of the few reported studies aimed at better understanding the substrate structural requirements necessary to induce the APEH switch from exopeptidase to endoprotease. Although further investigations are needed to address the actual mechanism adopted by the enzyme to recognize acetylated peptide or oxidized proteins, we hypothesize that this may take place through a conformational rearrangement, which could be dependent on the substrate’s physico-chemical and structural properties. Therefore, the substrates we have identified can be used to develop new probes useful for monitoring the endoprotease activity of APEH. For this purpose, starting from the GDF11[48-64]ox peptide, we are working on new fluorogenic substrates compatible with simple and fast assays that will facilitate the study of this promising diagnostic and/or prognostic biomarker involved in several human diseases.

## Figures and Tables

**Table 1:** Set of GDF11-related peptides containing methionine residues used as substrate models to evaluate the APEH endoprotease activity. Peptide sequences and their calculated (calcd.) and experimental (expm.) MWs, as estimated by LC-ESI-TOF mass analyses, are reported. Mass values were obtained from the deconvoluted mass spectra. Methionines (M) and residues replaced in the mutated peptides are highlighted in bold.

		Precursor Peptide		Oxidized Peptide	
ID PEPTIDE	Sequence	M.W. Calcd. (amu)	M.W. Expm. (amu)	M.W. Calcd. (amu)	M.W. Expm. (amu)
GDF11[48-64]wt	Ac-EY**M**F**M**QKYPHTHLVQQA-NH_2_	2191.02	2191.04	2223.02	2223.03
GDF11[48-64] K54E	Ac-EY**M**F**M**Q**E**YPHTHLVQQA-NH_2_	2191.97	2192.99	2223.97	2224.98
GDF11[48-64] Y49A	Ac-E**A**MF**M**QKYPHTHLVQQA-NH_2_	2098.99	2099.02	2130.99	2131.00
GDF11[48-64] F51A	Ac-EY**MAM**QKYPHTHLVQQA-NH_2_	2114.99	2115.01	2146.99	2147.00
GDF11[48-64] M50F51AA	Ac-EY**AAM**QKYPHTHLVQQA-NH_2_	2054.98	2055.06	2070.98	2071.00
GDF11[48-64] F51M52AA	Ac-EY**MAA**QKYPHTHLVQQA-NH_2_	2054.98	2055.03	2070.98	2071.00
GDF11[48-64]AA	Ac-EY**A**F**A**QKYPHTHLVQQA-NH_2_	2071.02	2071.00	-	-

**Table 2:** (**a**): GDF11-related peptides containing Met(O) residues used as substrate models to evaluate the APEH endoprotease activity. The fragments observed following APEH exposure are reported, together with sequences and amount of fragments generated calculated as percentage of the initial substrate. The experimental and calculated MWs (doubly charged ions) are also reported. Data are reported as the average of three independent experiments. SD, standard deviation. (**b**): Overall % of oxidized GDF11 peptides degraded upon exposure to APEH.

(a)					
ID PEPTIDE	aa Sequence	[M+2H]^+2^ calcd. *m*/*z*	[M+2H]^+2^ expm. *m*/*z*	%	±SD
GDF11[48-64] wt ox	Ac-EYM(ox)FM(ox)QKYPHTHLVQQA-NH_2_	1121.512	1121.522	49.6	3.4
	Ac-EYM(ox)FM(ox)QKYPHTHLVQ	1013.456	1013.465	16.4	1.7
	Ac-EYM(ox)FM(ox)QKYPHTHL	899.901	899.901	8.4	1.5
	Ac-EYM(ox)FM(ox)QKYPHTH	843.350	843.359	25.6	2.6
GDF11[48-64] K54E ox	Ac-EYM(ox)FM(ox)QEYPHTHLVQQA-NH_2_	1112.985	1112.998	81.3	4.9
	Ac-EYM(ox)FM(ox)QEYPHTH	843.820	834.835	18.7	1.2
GDF11[48-64] Y49A ox	Ac-EAM(ox)FM(ox)QKYPHTHLVQQA-NH_2_	1066.495	1066.508	78.3	3.9
	Ac-EAM(ox)FM(ox)QKYPHTHLVQ	967.440	967.451	10.2	1.1
	Ac-EAM(ox)FM(ox)QKYPHTH	797.337	797.346	11.5	1.3
GDF11[48-64] F51A ox	Ac-EYM(ox)AM(ox)QKYPHTHLVQQA-NH_2_	1074.496	1074.507	85.0	5.0
	Ac-EYM(ox)AM(ox)QKYPHTHLVQ	1039.465	1039.48	3.3	0.8
	Ac-EYM(ox)AM(ox)QKYPHTH	805.342	805.341	11.7	1.2
GDF11[48-64] M50F51AA ox	Ac-EYAAM(ox)QKYPHTHLVQQA-NH_2_	1036.494	1036.511	86.7	5.5
	Ac-EYAAM(ox)QKYPHTHLVQ	936.965	937.453	2.0	0.2
	Ac-EYAAM(ox)QKYPHTH	767.333	767.345	11.3	0.9
GDF11[48-64] F51M52AA ox	Ac-EYM(ox)AAQKYPHTHLVQQA-NH_2_	1036.494	1036.510	83.2	4.8
	Ac-EYM(ox)AAQKYPHTHLVQ	936.965	937.450	0.3	0.0
	Ac-EYM(ox)AAQKYPHTH	767.333	767.352	16.5	0.9
GDF11[48-65] M50M52AA	Ac-EYAFAQKYPHTHLVQQA-NH_2_	1125.071	1125.088	100	5.7
(**b**)					
**ID Peptide**		% Degradation *			
GDF11[48-64]wt ox		50.4			
GDF11[48-64]ox K54E		18.7			
GDF11[48-64]ox Y49A		21.7			
GDF11[48-64]ox F51A		15.0			
GDF11[48-64]ox M50F51AA		13.3			
GDF11[48-64]ox F51M52AA		16.8			
GDF11[48-64]wt		N.D. **			
GDF11[48-65]M50M52AA		N.D. **			

* Values are obtained as the sum of % reported in (a). ** N.D.: not degraded.

**Table 3:** Sequences of APEH endoprotease substrates with the indication of the amino acids whose oxidation has been experimentally verified (X(ox)), the residues more prone to oxidation (X), and the identified cleavage sites (**↓**). The subscript numbers indicate the position of the peptides respect to the full sequences. Bold indicates oxidized amino acids that are experimentally verified; background color indicates residues more prone to oxidation.

APEH Substrates	Sequences
Insulin A Chain	^1^GIVEQ**C(ox)C(ox)**A **↓** SV**C(ox)**SLY **↓** QLENY**C(ox)**N^21^
Bovine Serum Albumin	…^205^FGERALKAWSVARL **↓** SQKFPKAEF^227^… …^397^LGEYGFQNALIVRY **↓** TRKVPQVST^419^… …^493^DETYVPKAFDEKLF **↓** TFHADICTL^515^…
Amyloid-beta peptide	^1^DAEFRHDSGYEV**H**(ox) **↓H**(ox) **↓** QKLV**F**(ox) **↓** **F**(ox)AEDVGSNK^28^
GDF11[48-64]wt ox	^1^EY**M**(ox)F**M**(ox)QKYPHTH**↓** LVQQA^17^

## Data Availability

Original data supporting the findings described in this publication are available at CNR-IBBR and CNR-IBB.

## Supplementary Materials

The following supporting information can be downloaded at: https://www.mdpi.com/article/10.3390/ijms23010443/s1.
