# Supplementary material for: Oxidized Substrates of APEH as a Tool to Study the Endoprotease Activity of the Enzyme
Source: Int J Mol Sci. 2021 Dec 31;23(1):443. doi: 10.3390/ijms23010443 (PMC8745263; doi:10.3390/ijms23010443)
Supplement: Supplementary file 1 [file ijms-23-00443-s001.zip › ijms-1457809-supplementary.pdf]

SUPPLEMENTARY MATERIAL

Oxidized Substrates of APEH as a Tool to Study the Endoprotease Activity of the Enzyme

Annamaria Sandomenico [1,†], Marta Gogliettino [2,†], Emanuela Iaccarino [1], Carmela Fusco [2,3], Andrea Caporale [1,‡], Menotti Ruvo [1,*], Gianna Palmieri [2,*] and Ennio Cocca [2]

[1] Institute of Biostructure and Bioimaging, National Research Council (CNR-IBB), 80134 Napoli, Italy; annamaria.sandomenico@cnr.it (A.S.); emanuela.iaccarino@gmail.com (E.I.); andrea.caporale@cnr.it (A.C.)

[2] Institute of Biosciences and BioResources, National Research Council (CNR-IBBR), 80131 Napoli, Italy; marta.gogliettino@ibbr.cnr.it (M.G.); carmela.fusco@ibbr.cnr.it (C.F.); ennio.cocca@ibbr.cnr.it (E.C.)

[3] Department of Medicine and Health Sciences, University of Molise, 86100 Campobasso, Italy

* Correspondence: menotti.ruvo@unina.it (M.R.); gianna.palmieri@ibbr.cnr.it (G.P.)

† These authors contributed equally to this work.

‡ Present address: Institute of Crystallography, National Research Council (CNR-IC), 34149 Basovizza, (Italy).

Table S1: Percentage of differently oxidized species of the two main fragments obtained after by trypsin digestion of GDF11[48-65]ox.

		R.T. (min)	$MW_{calcd.}$ $[M+2H]^{2+}$	$MW_{expm.}$ $[M+2H]^{2+}$	%*
**Fragment 1**	Ac-EYMFMQK	-	509.745	-	-
	Ac-EYM(ox)FM(O)QK<br>Ac-EYM(O)$_2$FMQK<br>Ac-EYMFM(O)$_2$QK	9.53	525.745	525.712	24.3
	Ac-EYM(O)FMQK or<br>Ac-EYMFM(O)QK	-	517.745	-	-
**Fragment 2**	YPHTHLVQQA-NH$_2$	8.35	596.802	596.815	75.6
	YPH(O)TH(O)LVQQA-NH$_2$<br>YPH(O)$_2$THLVQQA-NH$_2$<br>YPHTH(O)$_2$LVQQA-NH$_2$	-	612.802	-	-
	YPH(O)THLVQQA-NH$_2$<br>YPHTH(O)LVQQA-NH2	-	604.802	-	-

The % are obtained from the peak areas measured in the Extracted Ion Chromatograms (EIC) of the doubly charged ions of each potential species obtainable for Fragment 1 and Fragment 2.

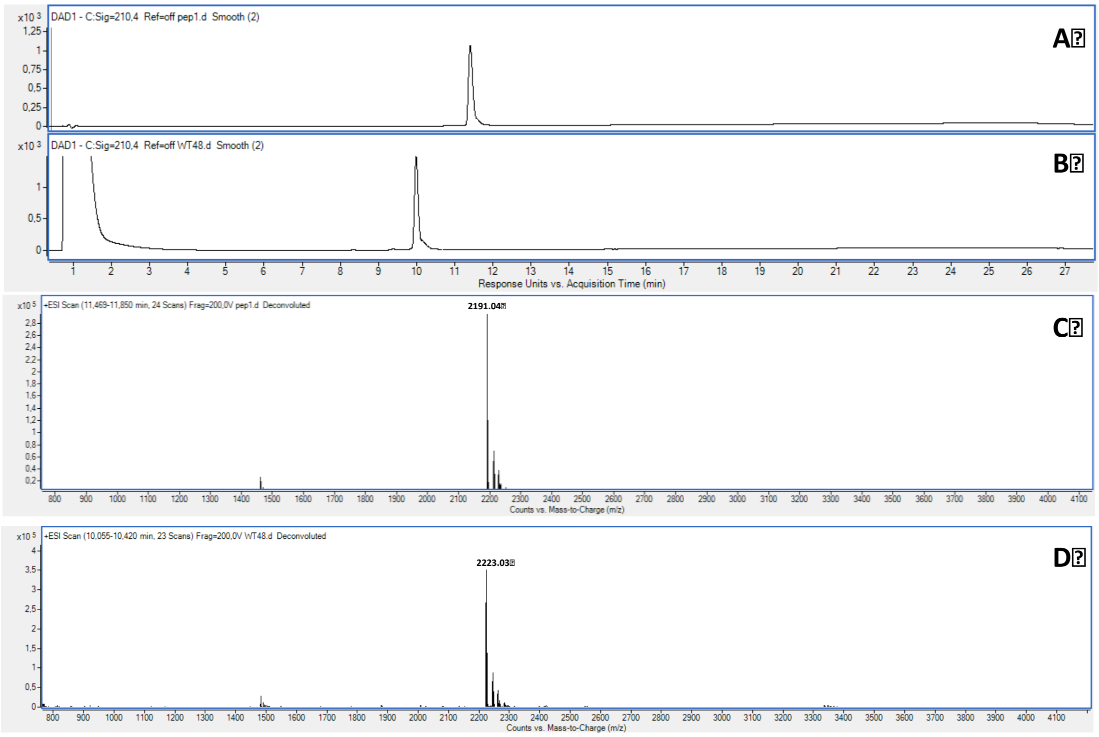

Figure S1. Oxidation reaction of GDF11[48-64]wt with hydrogen peroxide monitored by LC-ESI-TOF-MS (LC-MS) analysis. RP-HPLC profile of GDF11[48-64]wt before (**A**) and after (**B**) oxidation. Deconvoluted mass spectra of GDF11[48-64]wt before (**C**) and after (D) oxidation. The oxidation reaction of GDF11[48-64]wt was performed by incubating 1 µmol of peptide (2 mg) with 3% H_2O_2 in water for 20 min at room temperature. After incubation, the samples were collected and analyzed by LC-MS.

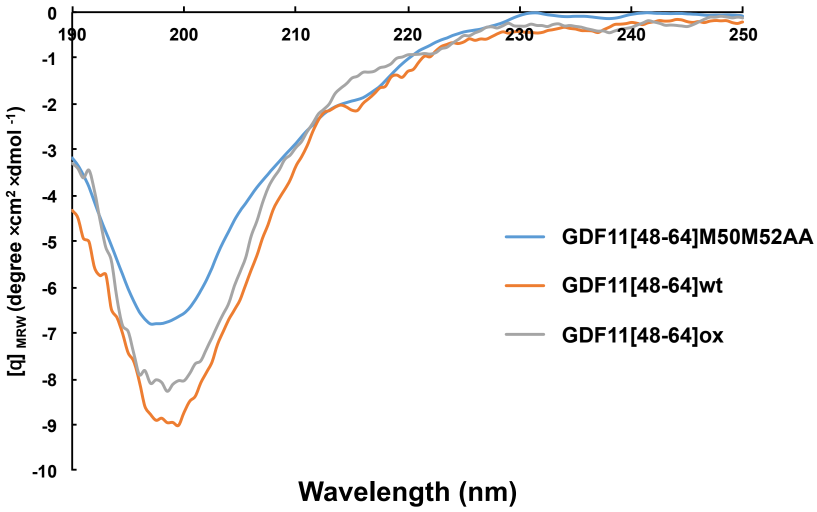

Figure S2. Overlaid CD spectra of peptides of GDF11[48-64]wt, GDF11[48-64]ox and GDF11[48-64]M50M52AA at 50 µM in water.

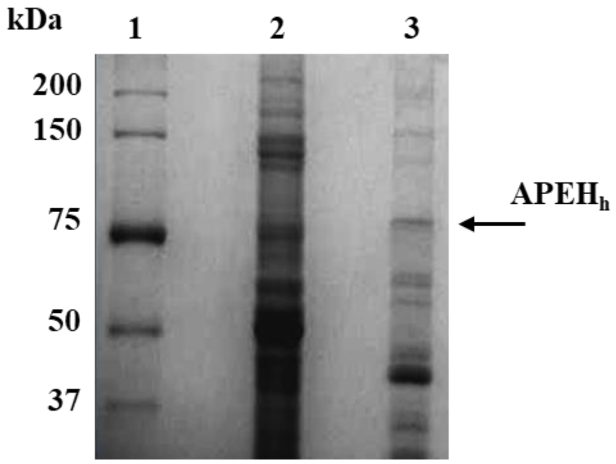

Figure S3. SDS-PAGE of the partially purified APEH from human RBC. Lane 1: molecular weight marker. Lane 2: protein fractions after DEAE chromatography. Lane 3: partially purified $APEH_h$ after gel filtration chromatography on Superdex 200 column. Protein bands were detected by Coomassie blue staining.

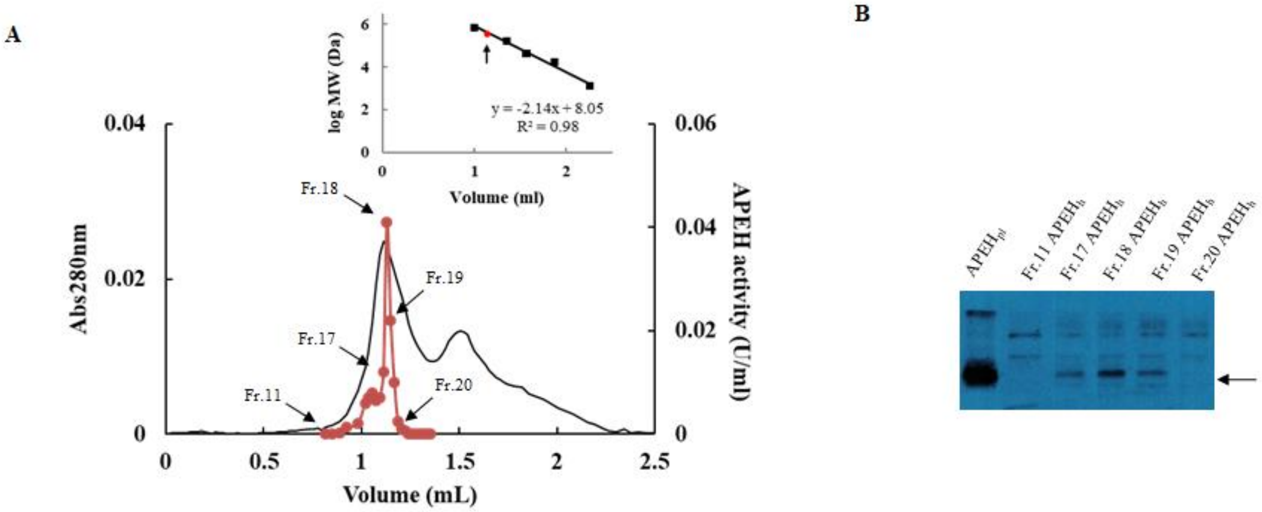

Figure S4. (**A**) Gel filtration chromatography on a Superdex 200 column of APEH from human RBC. The absorbance was measured at 280 nm. The profile in red represents the APEH activity monitored for each eluted fraction with the specific substrate Ac-Met-AMC. *Insert*: Calibration curve of the gel filtration column built using the following protein standards: Thyroglobulin (670 kDa), γ-globulin (158 kDa), Ovalbumin (44 kDa), Myoglobin (17 kDa), Vitamin B12 (1.35 kDa). (**B**) Immunoblotting analysis of gel filtration fractions using antiserum raised against human APEH. The commercially available APEH from porcine liver ($APEH_{pl}$) was used as control. The results are representative of three independent experiments on three different protein preparations.

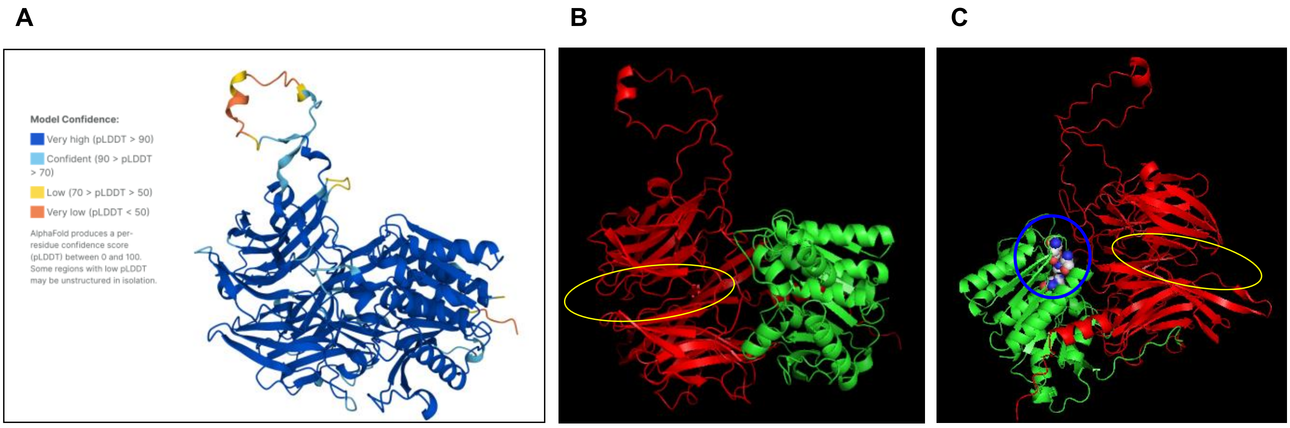

Figure S5. Structure of human APEH as determined by the server AlphaFold. (**A**) Structure model of human APEH, residues 1-732, with the confidence parameters reported on the left. (**B**) The same model as in A**,** where the bi-lobate structure is evidenced. The N-terminal β-propeller domain is coloured in red while the C-terminal hydrolase domain is in green. The tunnel in the β-propeller domain is highlighted in yellow. (**C**) The model is turned 180° and shows again the β-propeller tunnel (yellow) and the catalytic site made of S587, D675, H707, highlighted in blue.
